# Anatomic study of the position of the mandibular canal and corresponding mandibular third molar on cone-beam computed tomography images

**DOI:** 10.1007/s00276-017-1928-6

**Published:** 2017-10-27

**Authors:** Liqun Gu, Chao Zhu, Kejia Chen, Xianchu Liu, Zhangui Tang

**Affiliations:** 1 School of Stomatology, Central South University, Changsha, 410008 Hunan People’s Republic of China; 2 Department of Oral and Maxillofacial Surgery, Xiangya Stomatology Hospital, Central South University, Changsha, 410008 Hunan People’s Republic of China; 3 Department of Oral and Maxillofacial Radiology, Xiangya Stomatology Hospital, Central South University, Changsha, 410008 Hunan People’s Republic of China

**Keywords:** Mandibular third molar, Inferior alveolar nerve, Cone-beam computed tomography, Anatomic three-dimensional relationship

## Abstract

**Purpose:**

The positional relationship between the mandibular canal and corresponding third molars is a key anatomic factor of inferior alveolar nerve (IAN) injury. The aim of the present study is to classify the anatomic three-dimensional relationship between the mandibular third molar and the mandibular canal on cone-beam computed tomography (CBCT) images.

**Methods:**

This study used CBCT images to classify the positional relationship between the mandibular canal and corresponding third molars. CBCT images of 749 patients (1296 mandibular third molars) were analyzed to draw up a classification.

**Results:**

On a total of 1296 third molars, the mandibular canal relative to the roots of the mandibular third molar was on the apical side (88.1%), followed by the buccal side (7.9%), the lingual side (3.5%), and then between the roots (0.5%). Ninety-five (7.1%) third molars had a close relation with the mandibular canal, while 1201 (92.7%) third molars had no direct contact. The percentage of the mandibular canal contacts with the mandibular third molar was higher when the mandibular canal was lingually positioned.

**Conclusions:**

The anatomic structures of the mandibular third molar and the mandibular canal may be helpful to make adequate surgical planning to avoid or reduce nerve involvement.

## Introduction

Extraction of mandibular third molars is the most widely performed oral and maxillofacial surgery. Neurological injury is a rare but serious complication undergoing the operation. The incidence of inferior alveolar nerve (IAN) injury ranges from 0.4 to 6% [3, 4, 30]. Damage to IAN occurs most frequently when the mandibular third molar has a close relationship with the mandibular canal [29]. Imaging examination is the first step to assess the risk of IAN injury before operation.

The presence of certain radiographic signs on panoramic radiograph indicates a raised risk of IAN involvement. However, panoramic radiograph is observed in two dimensions. There are some overlapping images which make it hard to precisely judge the positional relationship, especially in the buccolingual direction [19, 21, 27].

Recently, cone-beam computed tomography (CBCT) has been widely used in clinical work due to its three-dimensional capability. CBCT not only provides reconstruction images on axial, coronal, and sagittal sections, but also shows the three-dimensional structures of the teeth and surrounding tissues [1, 25]. The aim of our study is to classify the anatomic three-dimensional relationship between the mandibular third molar and the mandibular canal, which may give a guidance to draw up the surgical plan in case of postoperative complications.

## Materials and methods

The present anatomic study was approved by the Independent Ethics Committee of the Xiangya Stomatology Hospital of Central South University, People’s Republic of China. Informed consent was obtained from all individual participants included in the study. 749 patients who had a CBCT scan and had one or two mandibular third molars with fully formed roots from January 2016 to June 2016 were consecutively included in this study with no restriction of gender. The patients had a mean age of 37.5 ± 13.6 years (ranged from 18 to 78 years). 123 patients had one mandibular third molar and 626 patients had two mandibular third molars. Among these molars, 79 third molars were excluded due to (1) residual roots; (2) upward movement due to deficiency of the corresponding maxillary third molar; (3) mesial movement due to deficiency of the adjacent mandibular second molar; (4) fracture and benign or malignant tumor associated with the corresponding mandibular third molar; (5) acute or chronic inflammation in the mandibular third molar zone. Finally, 1296 third molars were enrolled in this study.

The images were acquired using a CBCT scanner (Planmeca, Finland) with the following technical parameters: 90.0–96.0 kV, 10.0–12.0 mA, scan time < 16 s, and FOV of 100 mm × 90 mm. Voxel size was 150 or 400 μm and slice thickness of axial images was 0.20 mm. Images were processed using Planmeca Romexis software (Planmeca, Finland) to create axial, coronal, and sagittal reformatted images. Subsequently, the images were analyzed by two clinical radiologists with experience in the field of oral and maxillofacial radiology to classify the three-dimensional relationship of the mandibular third molar and the mandibular canal. If the results were controversial, a senior professor would join the discussion and the final results would be confirmed by the three people.

**Fig. 1:**
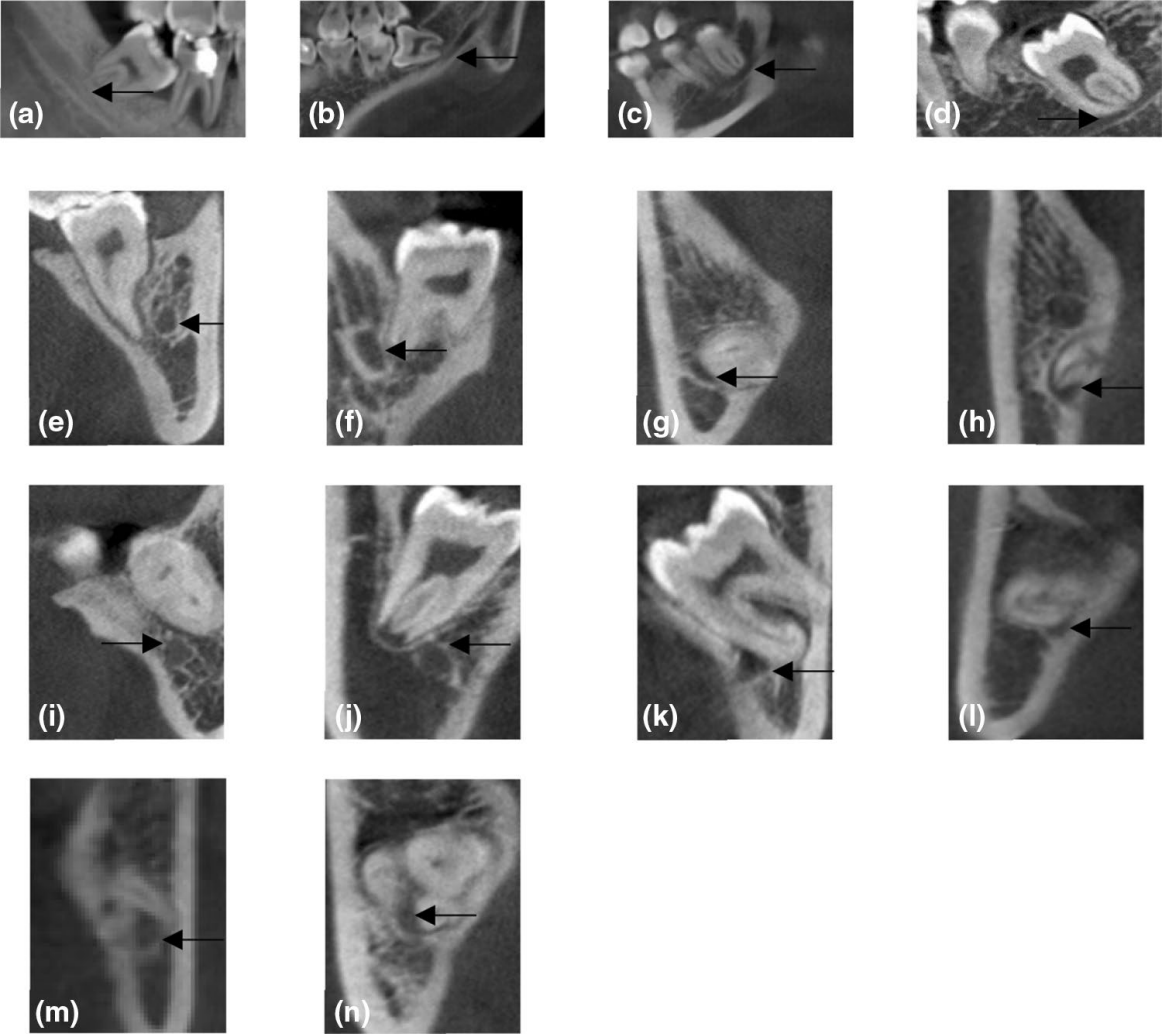
Classification of position and contact relation of the mandibular canal in relation to the mandibular third molar. Position of the mandibular canal in relation to the roots of the mandibular third molar was classified into four classes. Class I: apical position **a** no contact; **b** contact with a complete white line; **c** contact with a defective white line; **d** penetration of the mandibular canal. Class II: buccal position **e** no contact; **f** contact with a complete white line; **g** contact with a defective white line; **h** penetration of the mandibular canal. Class III: lingual position (**i**) no contact; (**j**) contact with a complete white line; **k** contact with a defective white line; **l** penetration of the mandibular canal. Class IV: interradicular position **m** contact with a defective white line; **n** penetration of the mandibular canal. No contact and contact with a complete white line of interradicular position were not found in this study (arrow indicates the mandibular canal)

Position of the mandibular canal relative to the roots of the mandibular third molar is defined as follows (Fig. 1a–n):

Class I: the mandibular canal locates on the apical side (apical position).

Class II: the mandibular canal locates on the buccal side (buccal position).

Class III: the mandibular canal locates on the lingual side (lingual position).

Class IV: the mandibular canal locates between the roots (interradicular position).

Contact relation of the mandibular third molar and the mandibular canal in each class is classified into four conditions (Fig. 1a–n).

1. The mandibular third molar has no contact with the mandibular canal.
2. The mandibular third molar contacts with the mandibular canal with a complete white line.
3. The mandibular third molar contacts with the mandibular canal with a defective white line.
4. The mandibular third molar penetrates the mandibular canal.

**Fig. 2:**
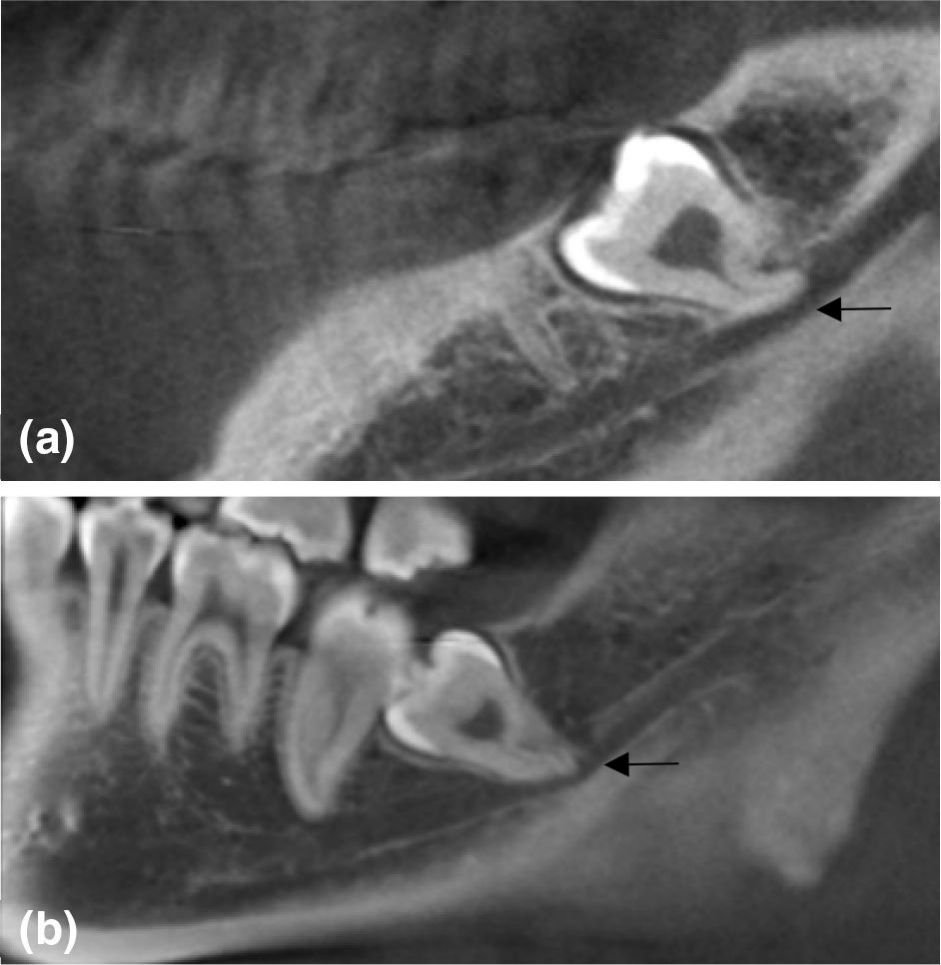
Vertical position of the mandibular third molar and the mandibular canal according to the penetration depth. Vertical status of the mandibular third molar and the mandibular canal was categorized in two conditions: **a** the root at the upper half of the mandibular canal;** b** the root at the lower half of the mandibular canal (arrow indicates the mandibular canal)

Vertical position of the mandibular third molar and the mandibular canal is categorized into two conditions according to the penetration depth of the roots: the root at the upper half of the mandibular canal; the root at the lower half of the mandibular canal (Fig. 2).

### Statistical analysis

The difference in the percentage of the three-dimensional position of the mandibular canal and corresponding third molars was evaluated by Pearson Chi-square test and Fisher’s exact test. *P* < 0.05 was regarded as significant. All statistical analysis was performed by SPSS software (Version 20.0.0).

## Results

In the assessment of the position of the mandibular canal in relation to the mandibular third molar on CBCT images, the inter-observer agreement was substantial (kappas of 0.779 for the position of the mandibular canal, and 0.71 for contact relation of the mandibular third molar and the mandibular canal).

The classification was drawn up according to (1) horizontal relationship between the mandibular third molar and the mandibular canal; (2) vertical relationship between the mandibular third molar and the mandibular canal; (3) the integrity of the mandibular canal wall.

Table 1 shows the anatomic position of the mandibular canal relative to the mandibular third molar in the whole study population. On a total of 1296 third molars, the mandibular canal in relation to the roots of the mandibular third molar was on the apical side (88.1%), followed by the buccal side (7.9%), the lingual side (3.5%), and then between the roots (0.5%). Table 2 shows that 95 (7.3%) third molars had a direct contact with the mandibular canal, while 1201 (92.7%) third molars had not. With respect to no contact with the mandibular molar, the mandibular canal tended to be on the apical side. When the mandibular canal contacts with the mandibular third molar, the percentage of the mandibular canal on the lingual side of the mandibular third molar was higher, compared to the “no contact” group (*p* < 0.05). Table 3 shows the different types of contact in Class II and Class III and no significant correlation was found (*p* > 0.05). Table 4 shows the vertical status of the mandibular third molar and the mandibular canal; Thirty-nine third molars were recorded and classified into two groups according to the penetration depth in the mandibular canal. No significant correlation was found (*p* > 0.05).

**Table 1:** Position of the mandibular canal relative to the mandibular third molar

	No contact (*n*)	Contact (*n*)		
		Contact with a complete white line	Contact with a defective white line	Penetration of the mandibular canal
Class I: the mandibular canal locates on the apical side	1110	12	10	10
Class II: the mandibular canal locates on the buccal side	82	5	5	10
Class III: the mandibular canal locates on the lingual side	9	4	17	16
Class IV: the mandibular canal locates between the roots	0	0	5	1

**Table 2:** Contact relation of the mandibular third molar and the mandibular canal in each class

	No contact (*n*, %)	Contact (*n*, %)	Total (*n*, %)
Classification			
Class I	1110 (85.6%)	32 (2.5%)*	1142 (88.1%)
Class II	82 (6.3%)	20 (1.5%)	102 (7.9%)
Class III	9 (0.7%)	37 (2.9%)**	46 (3.5%)
Class IV	0 (0%)	6 (0.5%)	6 (0.5%)
Total	1201	95	1296

*Significant difference in respect of “no contact” group (*p* < 0.05)

**Significant difference in respect of “no contact” group (*p* < 0.05)

**Table 3:** Different types of contact in Class II and Class III

	Class II (*n*, %)	Class III (*n*, %)	Total
Types of contact			
Contact with a complete white line	5 (8.8%)	4 (7.0%)	9
Contact with a defective white line	5 (8.8%)	17 (29.8%)	22
Penetration of the mandibular canal	10 (17.5%)	16 (28.1%)	26
Total	20	37	57

**Table 4:** Vertical status of the mandibular third molar and the mandibular canal

	Depth of penetration (*n*, %)		Total
	At the upper half	At the lower half	
Classification			
Class I	8 (21.6%)	2 (5.4%)	10
Class II	6 (16.2%)	4 (10.8%)	10
Class III	7 (18.9%)	9 (24.3%)	16
Class IV	1 (2.7%)	0 (0%)	1
Total	22	15	37

## Discussion

Removal of the mandibular third molar is one of the most common oral and maxillofacial operations. IAN injury is a serious complication after extraction of the mandibular third molar, affecting the function of the stomatognathic system and the quality of life of patients. The horizontal and vertical positions of the mandibular canal and corresponding third molars is a key anatomic factor of IAN injury [13, 18, 23]. Pre-operational analysis and evaluation are helpful to make a reasonable surgical management to avoid or reduce complications occurrence.

Panoramic radiography is routinely performed in clinical practice before extraction of the mandibular third molar to evaluate the risk of IAN injury [27]. Some radiographic features indicate that there is an increased risk of nerve damage associated with removal of the corresponding mandibular third molar [5, 13, 26]. However, it is not accurate to evaluate the relationship between the mandibular third molar and the mandibular canal in the buccolingual direction [19, 21, 27].

In this study, 95 (7.3%) third molars had a close relationship with the mandibular canal. Of these cases, a higher percentage was observed when the mandibular canal was on the lingual side of the mandibular third molar (*p* < 0.05). The previous study demonstrated that there is an increasing potential of IAN injury when the mandibular canal is situated lingually [9]. In addition, the mandibular third molar had a higher possibility to locate on the lingual side of the jaw [7]. We may hypothesize that lingually positioned mandibular canal is more likely to contact with the mandibular third molar due to insufficient space, as well as interradicular positioned mandibular canal. Although the prevalence of IAN injury is low, regarding a large number of population in China, the absolute number of possible nerve damage is significant. To reduce the risk of IAN injury, several operation techniques have been proposed.

The technique of coronectomy was to separate the crown completely while retaining the roots in the alveolar fossa. The remaining roots lied at least 3 mm below the crest of alveolar bone and the pulp was left untouched [20]. After surgery, root remnants had the ability to migrate away from the mandibular canal due to new bone formation above the roots, which reduced the possibility of IAN injury undergoing a necessary second operation [6]. The movement of remaining roots reached high rate at 6 months after operation and gradually stopped at 12–24 months postoperatively [14]. Unless the patient became symptomatic, the root remnants were considered as healthy to some extent [24].

Orthodontic extraction referred to orthodontic method to tract mandibular third molars and extraction of the teeth were performed consequently when the roots were far away from the IAN canal [2, 17]. A high risk of periodontal defect of the adjacent second molar on the distal surface was associated with this method [22]. Time-consuming and costly were also the drawbacks of this method.

Sagittal split osteotomy was one of the most common operations of mandibular deformity. It was used to remove a lower third molar in close proximity to the mandibular canal [16, 28]. The advantage was that direct recognition of anatomic structures helped operators to protect IAN, while the disadvantage was that the operation was an extensive and complex procedure.

There were several randomized controlled trials of CBCT versus panoramic radiography to assess the impact on patient outcomes for neural injury. All showed that CBCT did not lead to reduction of nerve injury and other complications, but was superior in predicting the risk of IAN injury [8, 11, 12, 15]. Consequently, CBCT contributed to more adequate surgical managements [10].

This is a study of Chinese patients. Within the limitation of the present study, the findings may not be generalizable. In addition, prevalence of nerve damage according to the three-dimensional structures is not studied. Therefore, further investigations are needed.

Taken together, the mandibular third molar correlated with higher expectation of nerve involvement when the mandibular canal was lingually situated. Our finding may be helpful to make an adequate surgical plan to avoid or reduce nerve involvement.
